# Accuracy and confidence of Irish general practitioners in diagnosing skin disease in patients with darkly pigmented skin

**DOI:** 10.1002/ski2.465

**Published:** 2024-10-21

**Authors:** Rachel Rey, Eileen Duggan, Cathal O’Connor

**Affiliations:** ^1^ Medicine University College Cork Cork Ireland; ^2^ Dermatology South Infirmary Victoria University Hospital Cork Ireland; ^3^ INFANT Research Centre University College Cork Cork Ireland

## Abstract

Managing skin conditions in patients with darkly pigmented skin (DPS) can be challenging due to inadequate exposure to dermatology in DPS in clinical training. In this study, Irish GPs were less likely to correctly diagnose common skin conditions in patients with DPS (*p* < 0.001) and had lower confidence levels in diagnosis in DPS (*p* < 0.001). Lower diagnostic accuracy and confidence with common skin conditions in DPS in primary care may lead to misdiagnosis, suboptimal treatment and increased referrals to dermatology.

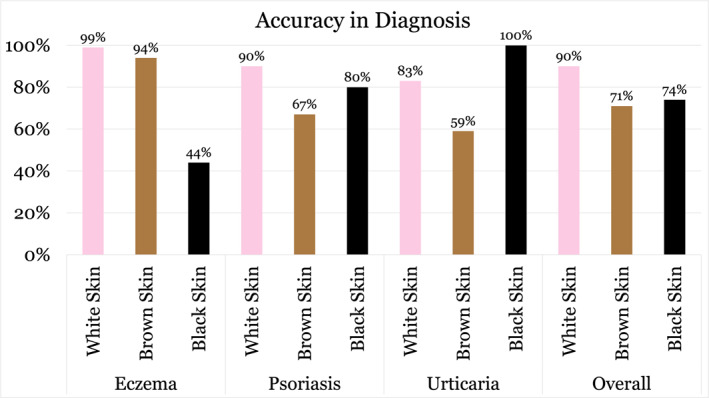

## 1 INTRODUCTION

Identifying and managing skin conditions in patients with darkly pigmented skin (DPS) can be difficult due to differing presentations of skin disease in DPS, inadequate representation of DPS in clinical training and insufficient exposure to patients with DPS in clinical practice.^1^ Reduced competence and confidence in managing skin disease in patients with DPS may lead to misdiagnosis, inappropriate or delayed treatment and increased referrals to secondary dermatology care.^1^ According to the 2022 Irish census, 12.6% of Irish people have DPS.^2^ Previous research has shown that half of the Irish dermatologists are ‘not confident’ or ‘not at all confident’ in diagnosing skin conditions and one‐third are ‘not confident’ or ‘not at all confident’ in managing skin conditions in patients with DPS.^3^ Skin complaints represent a significant proportion of presentations to general practitioners (GPs), and the vast majority of skin complaints are seen by GPs rather than dermatologists.^4^ The aim of this study was to assess the accuracy and confidence of Irish GPs in diagnosing skin conditions in DPS.

## 2 REPORT

Ethical approval was granted by the Clinical Research Ethics Committee of the Cork Teaching Hospitals [reference ECM 6 (u) 09/08/2022]. Irish GPs were invited to complete a questionnaire by email and by post. In total, 500 letters were posted and 250 emails were sent. The questionnaire assessed participants' demographics and training details and ability, confidence and perceived challenges in diagnosing common skin conditions in darkly and lightly pigmented skin (LPS) (Supplementary File 1 in Supporting Information S1). Images of atopic dermatitis/eczema, psoriasis and urticaria were presented in patients with white skin, brown skin, and black skin. Participants were asked to provide a diagnosis for each condition in each skin type and to rate their confidence in making the diagnosis.

There were 112 respondents (112/750, 14.9% response rate), of whom 60.7% (*n* = 68) were female, with a median age category of 41–50 years. Most (88.4%, *n* = 99) participants described their ethnicity as Irish, 5.4% (*n* = 6) as any other White background and 6.3% (*n* = 7) as Black, Indian/Pakistani/Bangladeshi, Asian or Hispanic. Most (91.1%, *n* = 102) were qualified GPs and 8.9% (*n* = 10) were senior GP trainees. The majority (59.8%, *n* = 67) said that 1%–5% of all patients attending their surgeries had DPS. There was no significant difference between urban and rural location in terms of estimated proportion of patients with DPS. The greatest challenges to diagnosis in DPS were rated as lack of training in dermatology in DPS by 55.4% (*n* = 62), lack of exposure to patients with DPS in 29.5% (*n* = 33) and lack of educational resources in dermatology in DPS by 15.2% (*n* = 17). In terms of training, 93% (*n* = 104) of participants reported that lighter skin tones were over‐represented during medical training. Only 41.1% (*n* = 46) and 19.6% (*n* = 22) had undertaken a formal dermatology placement at undergraduate and postgraduate levels, respectively. Most participants (59.8%, *n* = 67) strongly agreed or agreed to the prompt ‘I am aware of the nuances in the presentation of dermatological conditions in DPS’. Most (82.1%, *n* = 92) participants reported an experience of difficulty diagnosing a skin condition due to a patient's dark skin pigmentation. When asked how likely they are to refer a patient with DPS to secondary dermatological care, 42% (*n* = 47) said they were more likely, 56% (*n* = 63) said equally likely and 1.8% (*n* = 2) said less likely than patients with LPS. Most (89.3%, *n* = 100) would have liked the option of further training on dermatology in DPS, and only 22.3% (*n* = 25) had received teaching regarding the dermatology of DPS. Preferred methods for further teaching were face‐to‐face lectures/presentations (57.1%, *n* = 64), online learning modules (25%, *n* = 28) and webinars (13.4%, *n* = 15).

**FIGURE 1:**
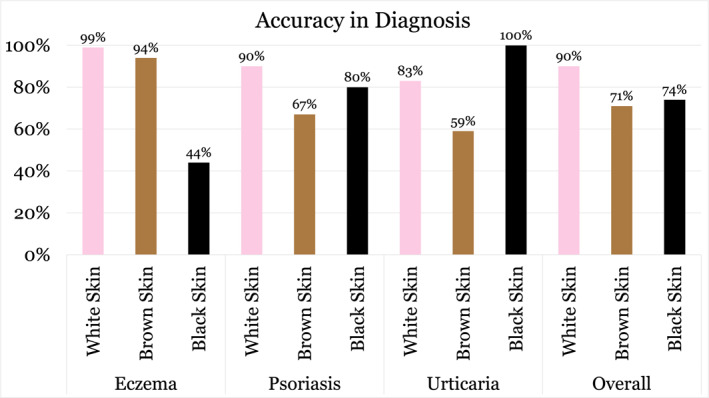
Accuracy of Irish GPs in the diagnosis of eczema, psoriasis, urticaria, etc., in white skin, brown skin and black skin (*n* = 112). GPs, general practitioners.

**FIGURE 2:**
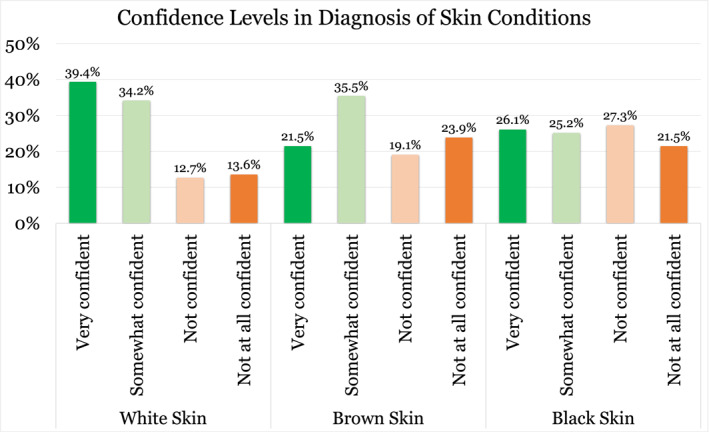
Confidence of Irish GPs in diagnosing common skin conditions in white skin, brown skin and black skin (*n* = 112). GPs, general practitioners.

Participants were significantly less likely to correctly diagnose common skin conditions in patients with DPS compared to LPS (*p* < 0.001). The average correct score was 90% for white skin , 71% for brown skin, and 74% for black skin (Figure 1). For eczema, 99.1% (*n* = 111) correctly diagnosed the condition in white skin, 93.8% (*n* = 105) in brown skin and only 43.8% (*n* = 49) in black skin. For psoriasis, 90.2% (*n* = 101) correctly diagnosed the condition in white skin, 67% (*n* = 75) in brown skin and 80.4% (*n* = 90) in black skin. For urticaria, 83% (*n* = 93) correctly diagnosed the condition in white skin, 58.9% (*n* = 66) in brown skin and 100% (*n* = 112) in black skin. Confidence levels in diagnosing these conditions were also significantly reduced in DPS (*p* < 0.001), with only 22.7% feeling ‘very confident’ when diagnosing DPS compared to 39.4% when diagnosing LPS (Figure 2). All (100%, *n* = 112) the respondents correctly identified dermatosis papulosa nigra. There was no significant difference according to years of experience, previous dermatology placements, number of patients seen per day or practice location in terms of diagnostic ability or confidence levels.

## 3 DISCUSSION

This study shows that Irish GPs have lower diagnostic accuracy and lower confidence levels in diagnosing and managing common skin conditions in DPS. This is consistent with previous work examining confidence in Irish and Australian dermatologists in diagnosing and managing skin conditions in patients with DPS.^3^, ^5^ This discrepancy in confidence between skin types is important as patients with DPS are known to have worse outcomes from dermatologic disease including melanoma.^6^ Almost half of the GPs stated that they are more likely to refer patients with skin complaints to secondary dermatology care, which has implications for dermatology service provision and resources. Only two in five GPs in this study had undertaken an undergraduate placement in dermatology, and only one in five had worked in dermatology, highlighting the lack of exposure of Irish GPs to dermatology training. Given the importance of racial equity in medicine,^7^ the availability of clinical images highlighting the appearance of dermatoses in DPS has increased. Resources to improve education in SOC include the Skin Diversity Subcommittee of the British Association of Dermatologists, Skin Deep (https://dftbskindeep.com/) and the Skin of Colour Society (https://skinofcolorsociety.org/). GPs stated that face‐to‐face lectures/presentations were the preferred method for further teaching. Although it can be hard to schedule teaching for busy GPs with demanding requirements for continuing professional development across a range of specialities, dermatologists should provide education on the subtleties of diagnosis and management of dermatoses in patients with DPS.

Strengths of this study include the assessment of accuracy as well as confidence in diagnosing skin conditions in DPS, the high number of responses, and the demographic representation of Irish GPs achieved. Limitations of this study include the low prevalence of DPS in the Irish population, potentially limiting the extrapolation of these results to regions with more patients with DPS. Diagnostic accuracy was based on a single image of a condition in different skin types, so the accuracy rate may have been different if more pictures had been provided. Although there was an adequate sample size to achieve statistically significant results for the main outcomes, the small numbers in subcategories meant that more detailed statistical analysis was not possible.

Lower diagnostic accuracy and confidence with common skin conditions in DPS in primary care may lead to misdiagnosis, suboptimal treatment and increased referrals to dermatology. The low levels of formal dermatology placements and training are concerning, highlighting the urgent need for improved training and education.

## CONFLICT OF INTEREST STATEMENT

None to declare.

## AUTHOR CONTRIBUTIONS

**Rachel Rey**: Conceptualization (equal); data curation (equal); formal analysis (equal); funding acquisition (equal); investigation (equal); methodology (equal); project administration (equal); resources (equal); software (equal); supervision (equal); validation (equal); visualization (equal); writing—original draft (equal); writing—review and editing (equal). **Eileen Duggan**: Conceptualization (equal); data curation (equal); formal analysis (equal); funding acquisition (equal); investigation (equal); methodology (equal); project administration (equal); resources (equal); software (equal); supervision (equal); validation (equal); visualization (equal); writing—original draft (equal); writing—review and editing (equal). **Cathal O’Connor**: Conceptualization (equal); data curation (equal); formal analysis (equal); funding acquisition (equal); investigation (equal); methodology (equal); project administration (equal); resources (equal); software (equal); supervision (equal); validation (equal); visualization (equal); writing—original draft (equal); writing—review and editing (equal).

## ETHICS STATEMENT

Ethical approval was provided by the Clinical Research Ethics Committee of the Cork Teaching Hospitals reference ECM 6 (u) 09/08/2022.

## PATIENT CONSENT

Informed consent was obtained from all study participants.

## Supporting information

Supporting Information S1

## Data Availability

Data are available on request from the corresponding author.
