# Supplementary material for: Accuracy and confidence of Irish general practitioners in diagnosing skin disease in patients with darkly pigmented skin
Source: Skin Health Dis. 2024 Oct 21;4(6):e465. doi: 10.1002/ski2.465 (PMC11608903; doi:10.1002/ski2.465)
Supplement: Supplementary file 1 — Supporting Information S1 [file SKI2-4-e465-s001.pdf]

Supplementary File S1. Questionnaire used in the study.

Challenges of diagnosing and managing skin conditions in patients with darkly pigmented skin in primary care

Chief Investigator: Dr Eileen Duggan, Lecturer, Medical Education Unit, School of Medicine, UCC
Contact Number for Chief Investigator:

Medical Student: Rachel Rey, 2nd Year Graduate Entry Medical Student, UCC
Email Address: 116332286@umail.ucc.ie

1. Project Description
The purpose of this research study is to uncover the experiences and challenges of General Practitioners (GPs) in the recognition of skin conditions on different skin tones as well as identifying opportunities to enhance training in dermatology in darkly pigmented skin (DPS). One of the main challenges reported by GPs is a lack of training in dermatology, and specifically in patients with DPS. This may lead to suboptimal knowledge and confidence for GPs, and thus poorer patient outcomes. For instance, common skin conditions such as eczema, acne vulgaris, and psoriasis present differently on DPS, however this is not clearly addressed in GP training. It will inquire about GPs ideas for improved training in dermatology in DPS in the future.

2. Why have I been chosen?
You have been asked to take part in this project because you are a practicing GP who has experience with patients presenting with skin conditions. Your valuable perspective and experiences are required for the purposes of this study and we would greatly appreciate your participation. Consent is implied from completion and submission of the questionnaire.

3. What will it involve?
You will be asked to provide background information on yourself and to complete a short questionnaire of 5 minutes duration. The questionnaire will be made available through a QR code which will give you access to the information sheet and questionnaire on a Google Form. When you are finished, you can submit the Google Form online. You will not be asked to provide your name or contact information on the forms; no personally identifiable information will be collected.

4. What will happen to the information collected?
Your information will be encrypted and stored on a password protected computer accessible only to the study team. Your information will be used in this study only and completed questionnaire will be discarded after 10 years.

5. Will the information I give be confidential?
This data will be analysed and presented in the medical student's final year research project report, however no personally identifiable information to you, will be analysed or included in the findings. If an opportunity arise to publish this data in a research journal, again all data will be kept anonymous. Electronic copies of questionnaires will be stored securely in the Chief Investigator's office in the Medical Education Dept.

This study has been approved by the Clinical Research Ethics Committee of the Cork Teaching Hospitals, Lancaster Hall, 6 Little Hanover Street, Cork, 021 490191

General Practice Information

Gender

- ◯ Female
- ◯ Male
- ◯ Other

Age

- ◯ 20-30
- ◯ 30-40
- ◯ 50-60
- ◯ >60

Place of work

- ○ Urban
- ○ Rural
- ○ Mixed (Urban and Rural)

Ethnicity

- ○ White
- ○ Black
- ○ Asian
- ○ Hispanic
- ○ Native Hawaiian or Other Pacific Islander
- ○ Mixed: Black - White
- ○ Mixed: Asian - White
- ○ Mixed: Asian - Black
- ○ Mixed: Hispanic - White
- ○ Mixed: Hispanic - Black
- ○ Mixed: Asian - Hispanic

I see approximately

- ◯ 1-20 patients per day
- ◯ 20-30 patients per day
- ◯ 30-40 patients per day
- ◯ 40-50 patients per day
- ◯ 50+ patients per day

What percentage of patients you see have darkly pigmented skin?

- ◯ 1-5%
- ◯ 5-10%
- ◯ 10-20%
- ◯ 20-50%
- ◯ >50%

Section 1: Perception of challenges

This section aims to uncover the challenges that GPs face in recognizing skin conditions in primary care.

Do you find it more difficult managing skin conditions in patients with darkly pigmented skin?

- ◯ Yes
- ◯ No
- ◯ Unsure

What do you think is the biggest challenge to looking after patients with darkly pigmented skin?

- ◯ Lack of training related to dermatology in darkly pigmented skin
- ◯ Lack of exposure to patients with darkly pigmented skin
- ◯ Lack of educational resources related to dermatology in darkly pigmented skin
- ◯ Other: ______________________

Section 2: Training Education

This section aims to assess the level of education in dermatology in GP training. Dermatology education is evaluated on a general level as well as across different skin tones. Strategies and interventions are suggested to ameliorate the training of GP in dermatology.

Definition of the term darkly pigmented skin (DPS) aka richly pigmented skin - this term refers to
- Individuals from racial groups with skin that is darker than that of Caucasians
- Examples: Asians (East Asians (Chinese, Korean, Japanese), South East Asians (Vietnamese, Malays, Singaporean, Thai, Cambodians), South Asians (Indian, Pakistanis, Bangladeshis), Africans, Hispanics (Mexican, Cuban, Puerto Rican, central and south American and others of Spanish descent), indigenous oceanic populations (Indigenous Australians, Samoan, Fijian, Cook Islands etc.)

Did you go on dermatology placement?

- ◯ Yes
- ◯ No

If yes, how long was your dermatology placement?

- ◯ 1-2 weeks
- ◯ 3-4 weeks
- ◯ >5 weeks
- ◯ N/A

Do you feel like you have been equally exposed to clinical images of lighter and darker skin tones?

- ◯ Yes
- ◯ No

If your previous answer was No, what skin tone was overrepresented?

- ◯ Lighter
- ◯ Darker
- ◯ N/A

Have you ever received specific formal training in dermatology in patients with DPS?

- ◯ Yes
- ◯ No

During your training, would you have liked more teaching in dermatology of DPS?

- ◯ Yes
- ◯ No

Would you be interested to participate in a course with skin conditions on DPS?

- ◯ Yes
- ◯ No

If yes, what teaching method would you prefer?

- ◯ Face-to-face lectures/presentations
- ◯ Online learning modules
- ◯ Webinars
- ◯ Online notes
- ◯ N/A

Section 3: Skin conditions on different skin tones

This section aims to assess the recognition of skin conditions different skin tones. It includes images of patients with varied skin presentations to evaluate whether the recognition of cutaneous diseases differ between white and DPS patients.

Definition of the term darkly pigmented skin (DPS) aka richly pigmented skin - this term refers to
- Individuals from racial groups with skin that is darker than that of Caucasians
- Examples: Asians (East Asians (Chinese, Korean, Japanese), South East Asians (Vietnamese, Malays, Singaporean, Thai, Cambodians), South Asians (Indian, Pakistanis, Bangladeshis), Africans, Hispanics (Mexican, Cuban, Puerto Rican, central and south American and others of Spanish descent), indigenous oceanic populations (Indigenous Australians, Samoan, Fijian, Cook Islands etc.)

Have you ever had difficulty identifying a skin condition due to the skin tone of patient?

- ◯ Yes
- ◯ No
- ◯ Maybe

I am aware of the nuances in presentation of medical conditions in darkly pigmented skin.

- ◯ Strongly agree
- ◯ Agree
- ◯ Neutral
- ◯ Disagree
- ◯ Strongly Disagree

What skin condition would you suspect if a patient present with this?

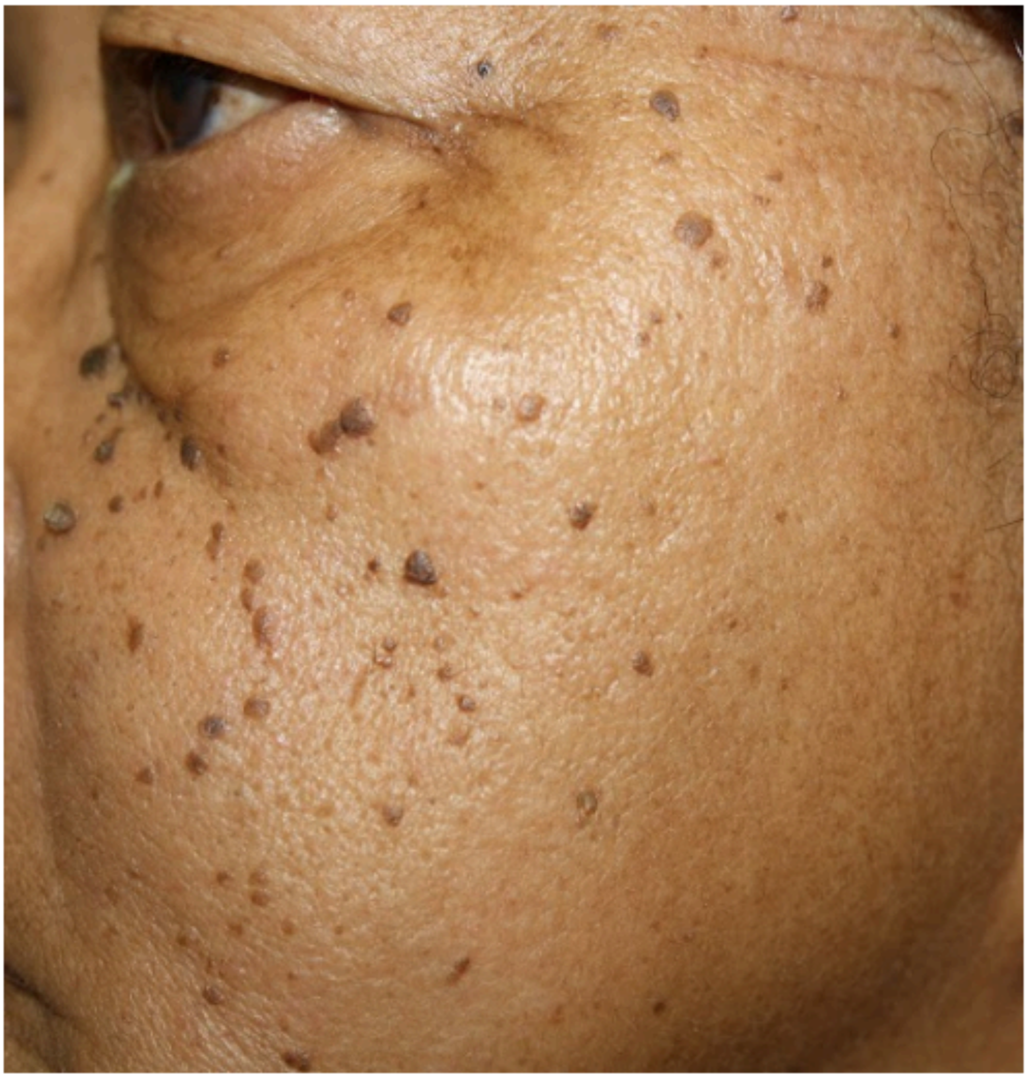

- Eczema
- Psoriasis
- Dermatosis Papulosa Nigra
- Urticaria
- Lichen Planus

How would you rate your confidence in diagnosing the patient above?

- Very confident
- Somewhat confident
- Not confident
- Not at all confident

What skin condition would you suspect if a patient present with this?

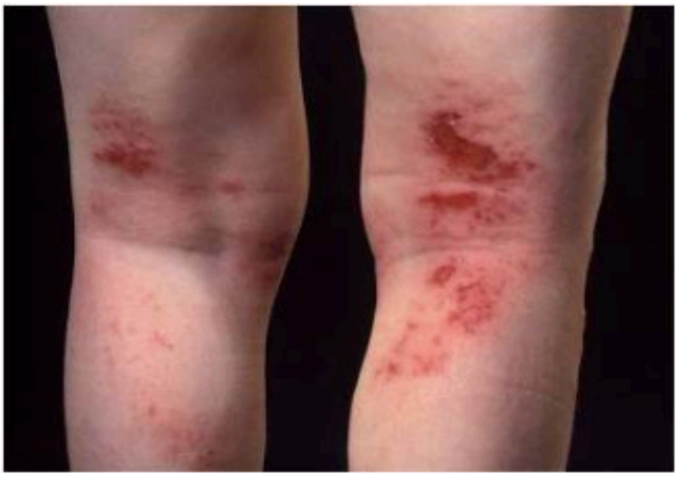

- Eczema
- Psoriasis
- Dermatosis Papulosa Nigra
- Urticaria
- Lichen Planus

How would you rate your confidence in diagnosing the patient above?

- Very confident
- Somewhat confident
- Not confident
- Not at all confident

What skin condition would you suspect if a patient present with this?

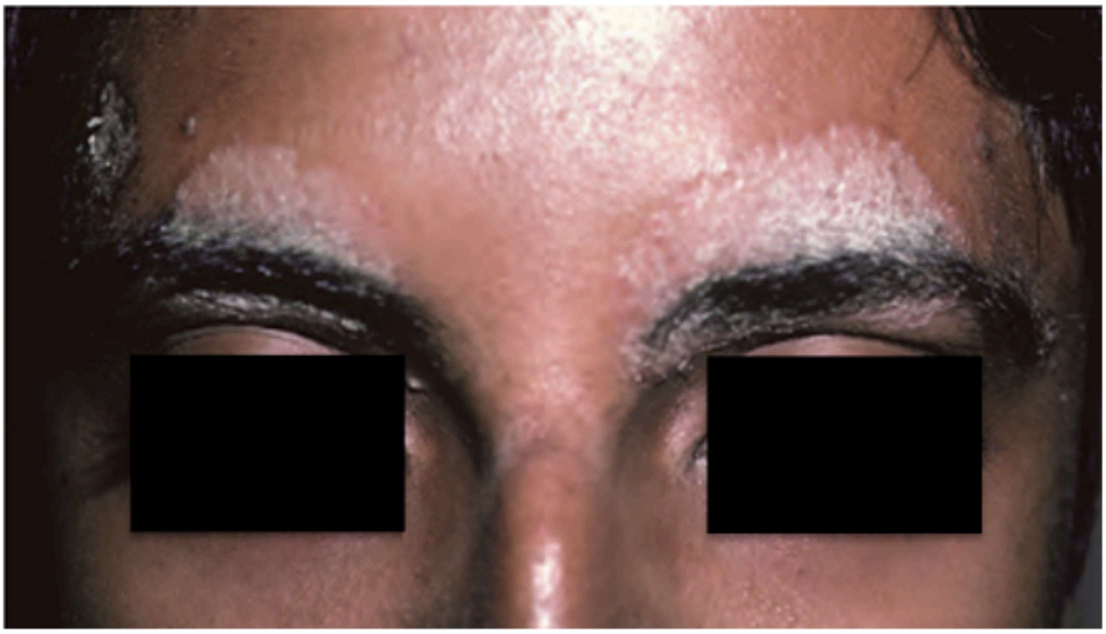

- Eczema
- Psoriasis
- Dermatosis Papulosa Nigra
- Urticaria
- Lichen Planus

How would you rate your confidence in diagnosing the patient above?

- ◯ Very confident
- ◯ Somewhat confident
- ◯ Not confident
- ◯ Not at all confident

What skin condition would you suspect if a patient present with this?

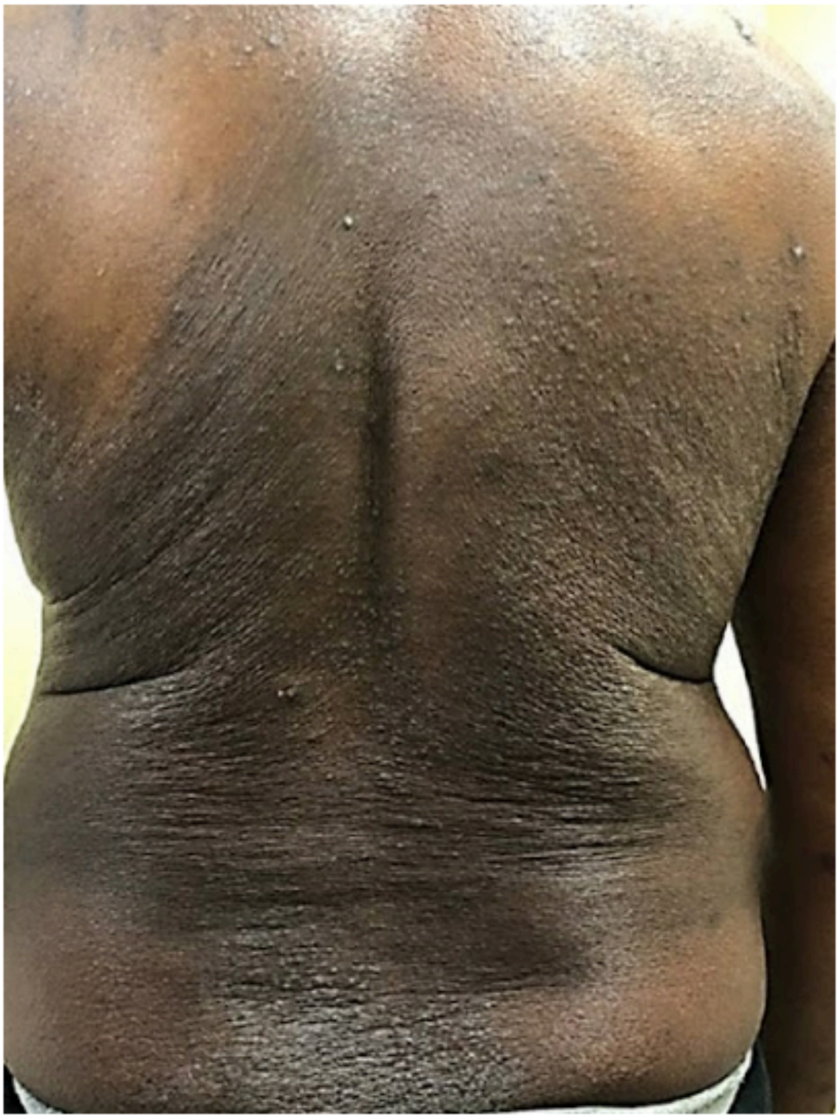

- Eczema
- Psoriasis
- Dermatosis Papulosa Nigra
- Urticaria
- Lichen Planus

How would you rate your confidence in diagnosing the patient above?

- ◯ Very confident
- ◯ Somewhat confident
- ◯ Not confident
- ◯ Not at all confident

What skin condition would you suspect if a patient present with this?

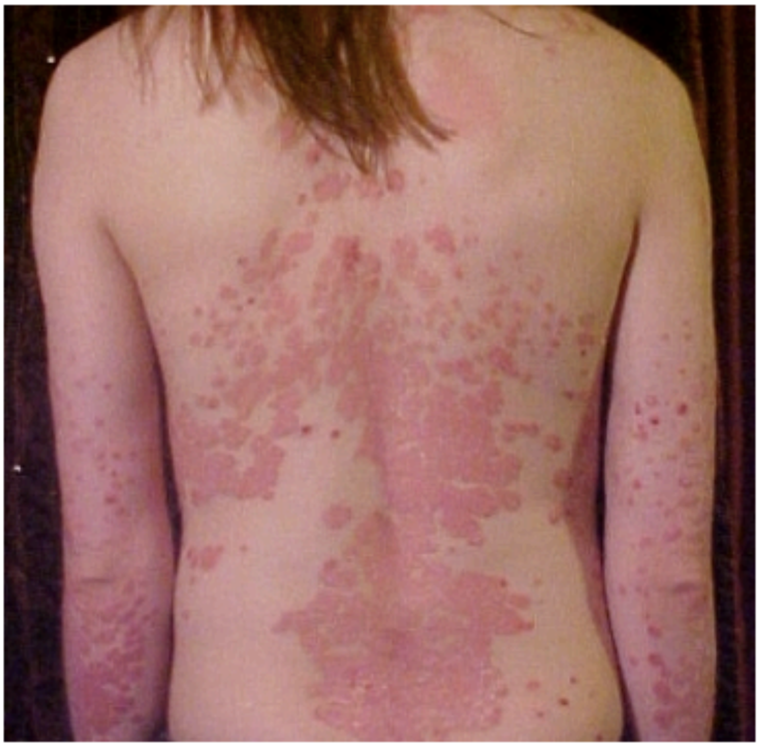

- Eczema
- Psoriasis
- Dermatosis Papulosa Nigra
- Urticaria
- Lichen Planus

How would you rate your confidence in diagnosing the patient above?

- Very confident
- Somewhat confident
- Not confident
- Not at all confident

What skin condition would you suspect if a patient present with this?

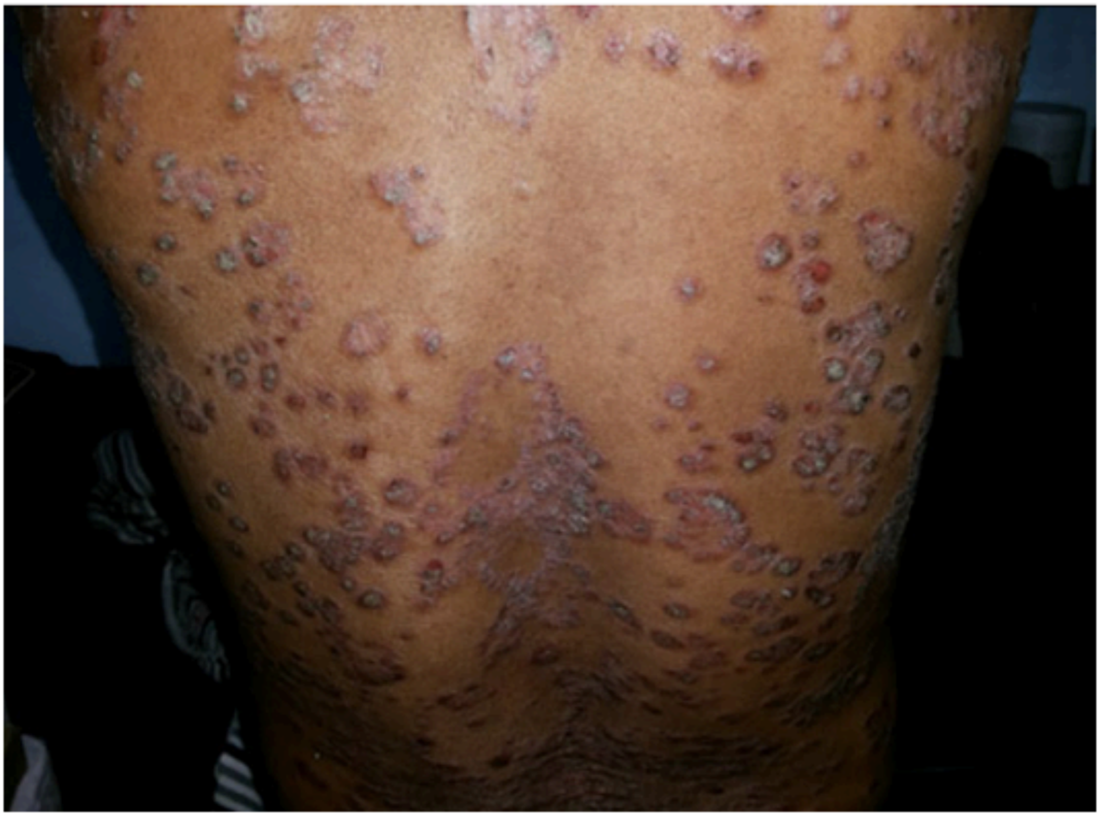

- Eczema
- Psoriasis
- Dermatosis Papulosa Nigra
- Urticaria
- Lichen Planus

How would you rate your confidence in diagnosing the patient above?

- ◯ Very confident
- ◯ Somewhat confident
- ◯ Not confident
- ◯ Not at all confident

What skin condition would you suspect if a patient present with this?

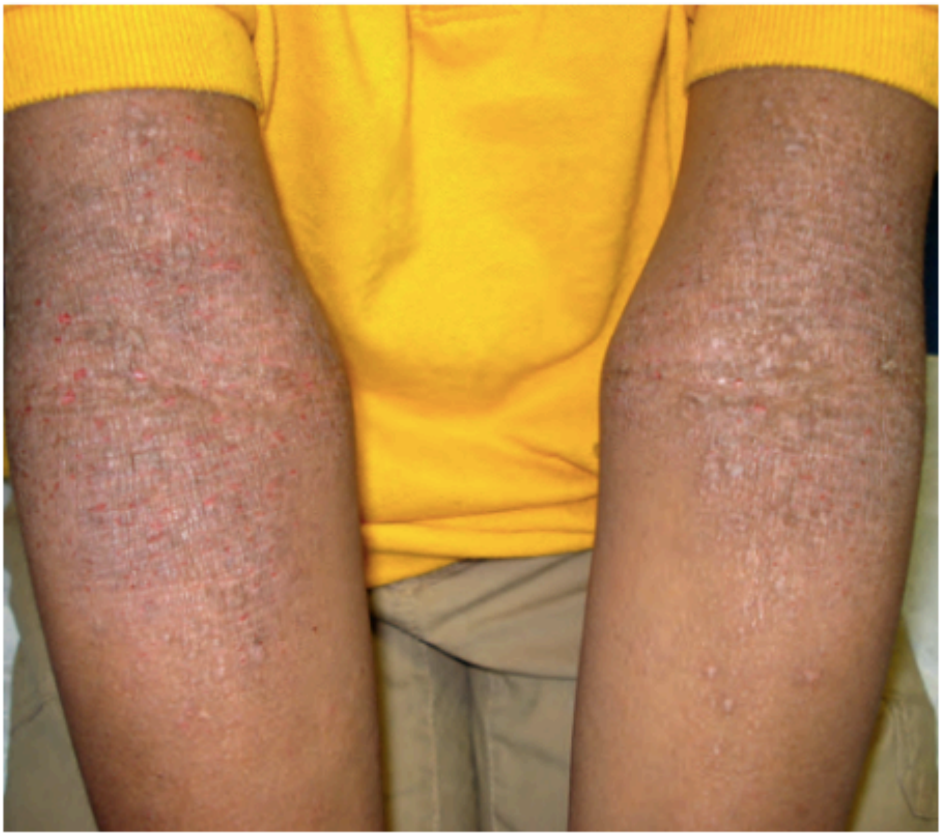

- ◯ Eczema
- ◯ Psoriasis
- ◯ Dermatosis Papulosa Nigra
- ◯ Urticaria
- ◯ Lichen Planus

How would you rate your confidence in diagnosing the patient above?

- ◯ Very confident
- ◯ Somewhat confident
- ◯ Not confident
- ◯ Not at all confident

Section 4: Confidence in skin condition recognition

This last section aims to evaluate the confidence of GPs when trying to identify dermatological conditions in general and across different skin tones.

Definition of the term darkly pigmented skin (DPS) aka richly pigmented skin - this term refers to
- Individuals from racial groups with skin that is darker than that of Caucasians
- Examples: Asians (East Asians (Chinese, Korean, Japanese), South East Asians (Vietnamese, Malays, Singaporean, Thai, Cambodians), South Asians (Indian, Pakistanis, Bangladeshis), Africans, Hispanics (Mexican, Cuban, Puerto Rican, central and south American and others of Spanish descent), indigenous oceanic populations (Indigenous Australians, Samoan, Fijian, Cook Islands etc.)

Are you confident in managing patients with dermatological conditions?

- ◯ Very confident
- ◯ Somewhat confident
- ◯ Not confident
- ◯ Not at all confident

How would you rate your confidence in looking after patients with DPS?

- Very confident
- Somewhat confident
- Not confident
- Not at all confident

How would you rate your confidence in diagnosing skin conditions in patients with skin of colour?

- Very confident
- Somewhat confident
- Not confident
- Not at all confident

Based on your clinical experience, did you ever consider contacting a specialist in the dermatology of DPS for your DPS patients?

- Every time
- Often
- Sometimes
- Never

Are you more likely or less likely to refer a patient with darkly pigmented skin to secondary dermatological care?

- ◯ Very unlikely
- ◯ Less likely
- ◯ More likely
- ◯ Very likely

What percentage of DPS patients with a skin condition would you refer to a specialist?

- ◯ Less than 50%
- ◯ 50 to 75%
- ◯ More than 75%
- ◯ 100%
